# Item analysis of G8 screening in uro-oncologic geriatric patients

**DOI:** 10.1007/s11255-023-03594-1

**Published:** 2023-04-17

**Authors:** J. Bouzan, S. Nellas, B. Stoilkov, P. Willschrei, M. Horstmann

**Affiliations:** 1 Department of Urology, Helios Hospital St. Josefshospital, Krefeld Uerdingen, Kurfuerstenstr. 69, 47829 Krefeld, Germany; 2 University of Duisburg-Essen, Hufelandstr. 55, 45147 Essen, Germany; 3 Department of Urology, Klinikum Guetersloh, Reckenberger Str. 19, 33332 Gütersloh, Germany; 4 Department of Geriatrics, Evang. Hospital Essen-Steele, Am Deimelsberg 34, 45276 Essen, Germany

**Keywords:** Geriatric screening, Geriatric assessment, G8 score, Geriatric urologic oncology, Nutrition, Item analysis

## Abstract

**Introduction/background:**

The G8 score is a widespread screening tool for geriatric frailty in oncology. The aim of this study was to evaluate the scores and relevance of G8 items in a standard screening of geriatric patients with uro-oncologic diseases to better understand the results of the assessment.

**Methods:**

Eighty-two consecutive uro-oncologic geriatric patients aged 75 years and older were evaluated. All patients underwent a G8 screening that consisted of 8 items. Patients with a G8 score above 14 were considered geriatric “fit”, while others were considered to be “frail”. Overall results and single item scores were evaluated. Clinical data were gathered from patients’ charts.

**Results:**

The mean age of the patients was 82 years (min. 75–max. 102). In 36 of the patients, the G8 score indicated “no-frailty”, and in 46 patients, the G8 score indicated “frailty”. The mean G8 score was 12.9 (min 4–max 17 pts). Item analysis revealed that points were most often lost in items H (polypharmacy), P (comparison of health status to peers) and Age. Fifty-nine, 56 and 52 patients lost points on item Age, item H and item P, respectively. In contrast, the majority of patients reached the maximum score for nutritional items [i.e., items A (food intake), B (weight loss) and F (body mass index (BMI))]. For item A, 73 patients reached the maximum score; for item B, 62 patients reached the maximum score; and for item F, 72 patients reached the maximum score. There were no differences in this distribution pattern when comparing tumour entities, sex, and patients with local vs. metastatic disease.

**Conclusion:**

The present study revealed a high percentage of suspicious test results. Potential reasons for these findings include the low threshold of the G8 overall score and the fact that in some items, points were easily lost. Modifications of the test should be considered.

## Introduction

**Fig. 1:**
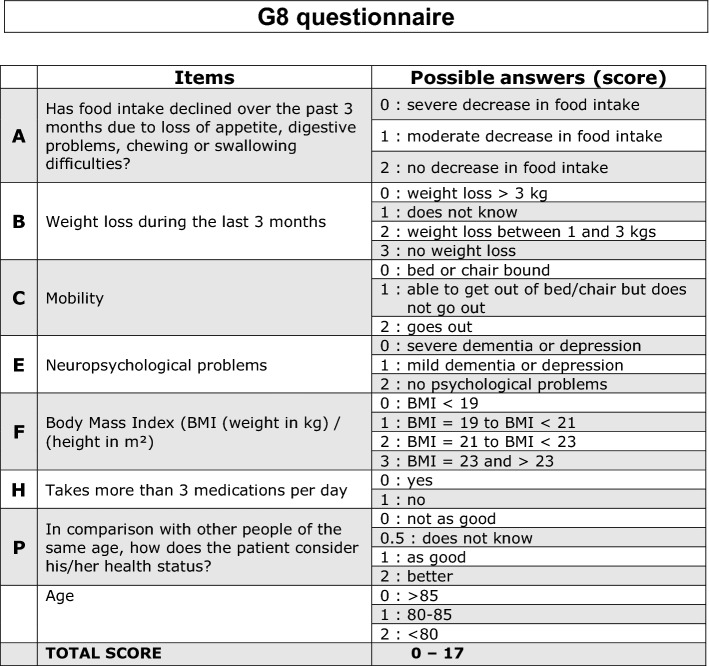
G8 score questionnaire

The G8 score is a geriatric screening tool that is frequently used and discussed in oncology [1, 2] and urologic oncology [3]. The questionnaire includes eight items (Fig. 1) [4]. The first seven items are taken from the MINI Nutritional Assessment (MNA) [5, 6], which itself consists of 6 items for screening and 12 items for assessment. Only the eighth item of the G8 score—which assesses age—is new. The G8 score is constructed like a check list, which makes it easy and quick to fill out within 5–8 min. The G8 score was first introduced by Bellera et al. [7], who validated it in 364 oncology patients who were older than 70 years of age and who had advanced disease prior to chemotherapy. All patients underwent a complete geriatric assessment (CGA) as a reference test for geriatric frailty. The sensitivity and specificity of the G8 score for geriatric frailty were above 85% and 65%, respectively, in their validation study.

Because it originates from the MNA, the G8 score mainly assesses nutritional aspects. Nutritional aspects are assessed by three of the eight items. Item A addresses food intake, item B assesses weight loss, and item F assesses body mass index (BMI) (Fig. 1). Other single items assess mobility (item C), neuropsychological restrictions (item E), polypharmacy (item H), age (AGE), and subjective health status relative to the respondent’s peers (item P) (Fig. 1). Each item is scored on a scale ranging from 0 to—depending on the item—1–3 points. The scores for all items are summed to obtain a final score. Patients with scores of at least 14 points (out of a maximum of 17 points) are considered geriatric “fit or healthy”, whereas patients with scores of 14 or less are considered “frail”. Patients with a G8 score of 14 or less points should undergo further geriatric evaluation. This is currently best ensured by a full comprehensive geriatric assessment (CGA) [8, 9].

In the past decade, the G8 score has proven to be a valuable screening tool in geriatric oncology [7, 10, 11]. In some studies, it has been proven to be of prognostic value prior to cancer treatment [2, 12, 13]. However, if it is used alone and out of context, it has some drawbacks. In addition to its low specificity [14], some geriatric impairments (e.g., cognition) are poorly assessed by the test [15], because it focuses on nutritional aspects. In urologic oncology, the G8 score is currently recommended for geriatric prostate cancer and muscle invasive bladder cancer patients by the International Society of Geriatric Oncology (SIOG), the European Society of Urology (EAU), and the European Society for Radio therapy and Oncology (ESTRO) [16–18]. To date, the G8 score has only been slightly validated and evaluated in urologic oncology. In the present study, we evaluated the results of G8 screening in a heterogeneous group of uro-oncologic patients with prostate, bladder, and renal cancer. We examined the items of the G8 score and evaluated which items individuals most often lost points on. The aim of the current study was to better understand the G8 score in uro-oncologic patients.

## Methods

Eighty-two consecutive patients aged 75 years or older who were admitted to the hospital were retrospectively included in the study. All patients were treated between 2019 and 2020 for uro-oncologic diseases at the Department of Urology of the St. Josefshospital in Krefeld Uerdingen, Germany. All patients routinely underwent G8 screening. Tests were administered at admission by urology residents (J. B., B.S., and S.N.). A G8 score ≤ 14 points was considered positive for geriatric “frailty”. Patients with a G8 score > 14 were classified as geriatric “fit”. The following patient data were collected from patients’ charts; age, sex, reason for admission, comorbidities, and BMI. In the present study, we first looked at overall test results at a cut-off of 14 points. Then, we performed a single item analysis to determine which items were associated with lost points. Statistical analyses were performed by Excel, Microsoft, 2016, Version 15.34. The study was performed in accordance with the ethical standards of the Medical Council of North Rhine Westphalia, Germany. The need for ethical approval was waived by the Medical Council due to the retrospective nature and anonymous evaluation of routine data (248/2020).

## Results

**Fig. 2:**
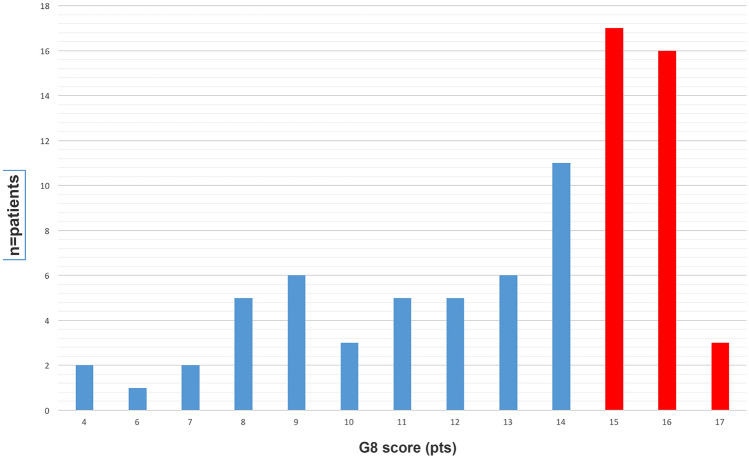
Overall G8 score test results of 82 patients. Blue depicts the number of patients with G8 scores indicating frailty, and red depicts the number of patients with a G8 score indicating no frailty

The mean age of the patients was 83 years (min. 75 years–max. 102 years). The patient characteristics are listed in Table 1. According to the G8 test results, 36 of the patients were considered geriatric “fit”, and 46 were considered “frail”. The mean G8 score of all patients was 12.9 (min 4–max 17 pts). G8 scores and the number of patients who were above and below the cut-off values are shown in Fig. 2. Three patients had a total score of 17 pts, sixteen patients had scores of 16 points, and 17 patients had scores of 15 points, indicating that these patients were “fit”. Among the patients who were considered “frail”, 11 patients had scores of 14 points. This group of patients was the largest group of patients classified as G8 “frail” (Fig. 2).

**Table 1:** Patient characteristics

	All
Patients (M/F), (*n* = pat.)	82 (63/19)
Age (mean years, min–max)	83 (75–98)
Prostate cancer (n=pat.)	31
Localized	23
Metastatic	8
Urothelial cancer (n=pat.)	45
Localized	35
Metastatic	10
Renal cancer (n=pat.)	6
Localized	4
Metastatic	2
G8 score [mean (min–max)]	12.9 (4–17)
BMI	25.8 (17–40)

**Fig. 3:**
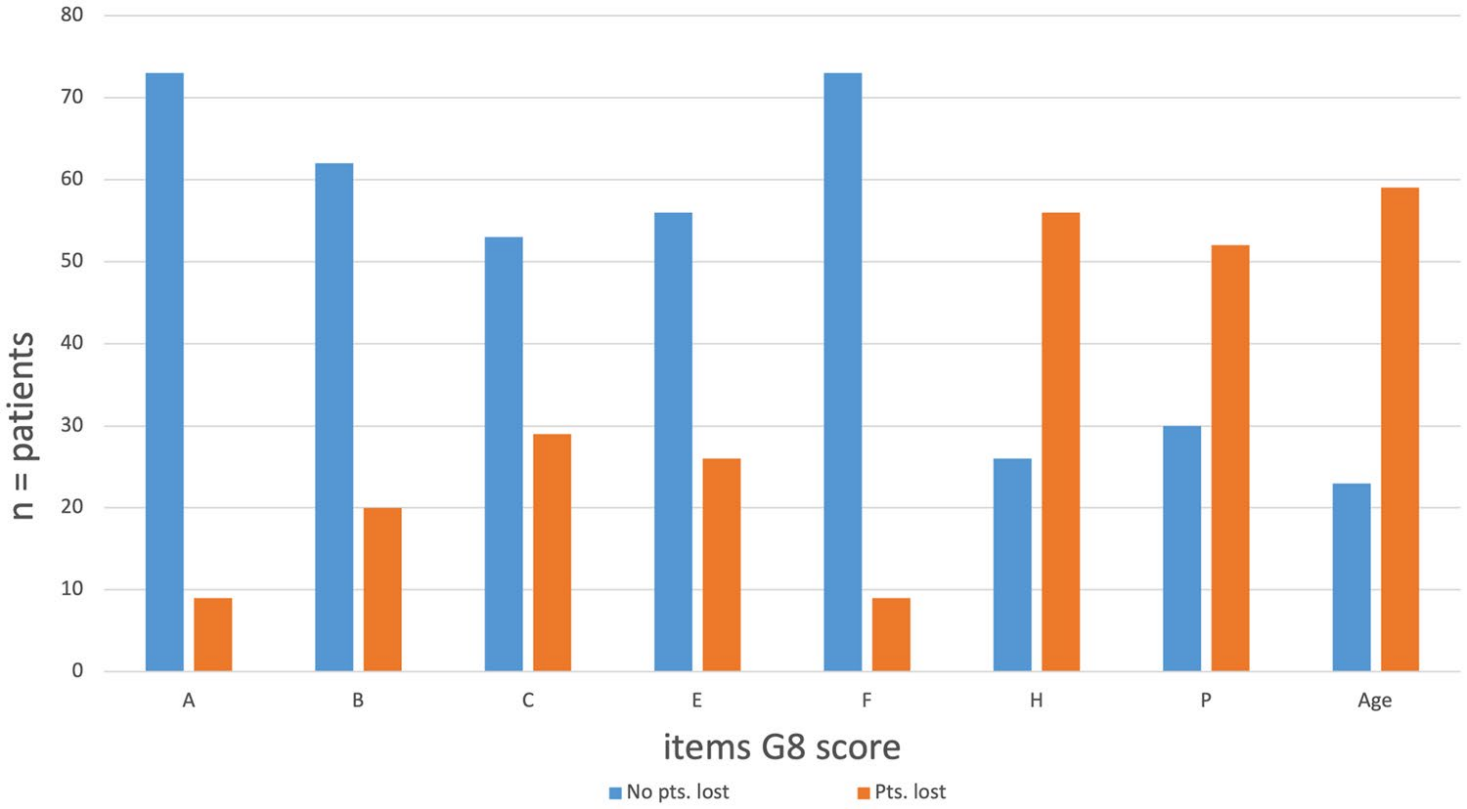
Item results of the G8 score of 82 patients. Blue depicts patients with a full item score, and red depicts patients who lost points in the item

The item analysis revealed that points were most often lost for item H (polypharmacy), item P (comparison of health status to peer), and item Age (Fig. 3). Most often, patients lost points (*n* = 59) on the item Age, losing 1 point due to an age above 80 years (*n* = 37) or 2 points due to an age above 85 years (*n* = 22). Additionally, 56 patients lost 1 point on item H (polypharmacy) due to taking more than 3 drugs. The third most common item for which patients lost points was item P (comparison health status to peers). For this item, 52 patients lost points in their subjective self-evaluation comparing their health status with peers; 30 patients lost 1 point on item P for feeling “as good as peers”, 6 patients lost 2 points for feeling “less good than peers”, and 1 patient lost 1.5 points for “not knowing”.

In contrast, the majority of patients reported the maximum scores for items A (food intake), B (weight loss), and F [body mass index (BMI)] (Fig. 3). All of these items assess nutritional aspects. For item A, 73 of the patients stated that they had “no changes in food intake”. Only 7 and 2 patients declared that they had “moderate” or “severe changes in food intake”, respectively. For item B (weight loss), the majority of 62 of the patients obtained a maximum score of 3 points by reporting “no change of their weight”. In this item, 3 patients lost 1 point due to a weight loss of 1–3 kg, 15 patients lost 2 points for “not knowing”, and 2 patients lost three points due to a weight loss of  > 3 kg. For item F (BMI), the majority of 72 of the patients had a BMI of  > 23 kg/m^2^. Six patients lost 1 point on item F due to a BMI of 21 to <23 kg/m^2^, three patients lost 2 points due to a BMI of 19 to < 21 kg/m^2^, and one patient lost 3 points due to a BMI of  < 19 kg/m^2^.

Additionally, for the remaining items, including items C (mobility) and E (neuropsychological problems), most patients had scored of 2 points. For item C (mobility), the majority of 50 of the patients had a score of 2 points for being able “to leave the house”. Nineteen patients lost 1 point for this item for being able “to stand up but not being able to leave the house”. Ten patients lost 2 points for being “restricted to bed or chair”. For item E (neuropsychological problems), the majority of 54 patients reported “no psychological problems”. Twenty patients lost 1 point on item E due to “mild dementia or depression”, and 8 patients lost 2 points because of “severe dementia or depression”.

Finally, we evaluated subgroups and compared whether the pattern of points lost differed between tumour entities, sexes, and patients with local vs. metastatic disease. This comparison revealed that in all subgroups, points were most often lost on subitems H, P and age rather than items A, B, C, E, and F (compared to Fig. 1). Differences between the patterns were not significant.

## Discussion

The G8 score is a widely discussed and recommended geriatric screening score not only in oncology [1, 2] but also in urologic oncology [4, 19, 20]. One of its drawbacks is its low specificity, with a high rate of patients who are G8 “frail” and require further investigation [11]. In urologic oncology, published clinical experiences of G8 score test results are scarce and often only report overall test results [4, 11, 21]. In the present study, we evaluated G8 score results in a consecutive group of uro-oncologic patients and performed an item analysis of the G8 score to better understand test results for everyday clinical practice. Our aim was to understand which items were associated with patients losing points and the underlying reasons.

First, our analysis confirmed the high number of G8 “frail” patients in our cohort. A total of 56% (*n* = 46) of patients were G8 “frail”, i.e., they had a G8 score of 14 or less points. This was true, even though 62 (75%) of the patients only had organ-confined disease. The results are similar to the literature published for urologic and other malignancies [10]. Ademi et al. reported that 81 out of 101 lung cancer patients who prospectively underwent G8 screening were G8 “frail” [13]. Souwer et al., who screened 132 patients older than 79 years with colorectal cancer by a G8 score, reported that 68 (50%) of the patients were positive for frailty according to the G8 score results [14].

As a next step, we performed a G8 score item analysis to better understand the high rate of G8 “frail” patients. This revealed that in our group of urooncologic patients, points were most often lost in the items of Age, polypharmacy (item H), and subjective comparison of health status to peers (item P). In particular, age proved to be of high relevance for the high number of G8 “frail” patients. The reason for this is that according to the G8 test design, points are systematically lost if the numeric age of the patients is above 80 years (− 1 point) or above 85 years (− 2 points). To give an example, this means that a patient at the age of 87 years, regardless of his biological age, systematically loses two points. As a consequence, he can only reach a maximum of 15 points in the overall G8 score. At a cut-off of 14 points, this is just one point above a test result, that is positive for frailty. Therefore, age is highly relevant for a huge number of positive test results.

Following item Age, polypharmacy (item H) was the second most frequent item on which score points were lost. The high number of points lost on that item is due to the threshold of only > 3 drugs for losing 1 point because of polypharmacy. Compared to other geriatric screening scores, this is low. The threshold of the ISAR Score, for example, is five drugs [11]. In the literature, these aspects were realized by Petit-Moneger et al. from Bordeaux. To further improve the specificity of the G8 score, they proposed increasing the threshold to six drugs [22]. The third most frequent item on which points were lost was item P. For this item, the patient’s subjective health status relative to peers is assessed. In our experience, the reason for the loss of points on this item was the subjective item design with wording that only “feeling better” than peers results in the maximum score. In our experience, patients were generally reluctant to claim that their personal situation was “better” than that of others and thus selected other response options. As a summary of these three discussed items, we found that the threshold for losing points on these items was very low and that losing points on them led to the high number of G8 “frail” patients.

In contrast to the above-mentioned items—Age, item H (polypharmacy), and item P (comparison of health status to peers)—the majority of patients lost no points on items A (food intake), B (weight loss), and F (BMI). Interestingly, these items are all related to nutritional aspects, which is consistent with the fact that the G8 score was derived from the Mini Nutritional Score (MNA) [5, 6]. We attribute the reason for these results to the fact that urologic malignancies, especially localized malignancies, do not lead to weight loss, difficulties in food intake or loss of appetite. This is in contrast to other malignancies in the digestive system, in which loss of appetite, difficulties of food intake, and weight loss are often the first symptoms of the disease. In urooncology, even patients with advanced and metastatic malignancies do not necessarily suffer from severe weight loss or difficulties in food intake. Patients with metastatic prostate cancer, for example, even regularly gain weight instead of losing weight because of androgen deprivation [3]. These considerations lead to uncertainty regarding whether these items are the best for geriatric screening in uro-oncologic patients [19].

Whereas nutritional aspects are assessed by three items of the G8 score, all other dimensions, including mobility (item C) and neuropsychology (item E), are addressed by only one item. It is important to note that, with respect to the validation of the latter items, several of them do not objectively measure facts but are based on subjective evaluations by both the patient and the interviewer. The neuropsychological item (item E), for example, does not include any objective measurement tools for cognitive disorders or depression. Rather, it depends on known diagnosis, the self-evaluation of the patient and/or the evaluation of the investigator. According to our experience, self-evaluation is difficult for patients. In clinical practice, it often results in a score of “no psychological problems” rather than a report of any neuropsychological deficits. Interestingly, in our recent study combining the G8 score with more objective Mini-Cog screening, we found that a significant number of patients with G8 overall tests indicating no frailty nevertheless have a Mini-Cog screening positive for cognitive disorders in a common screening population [15].

Altogether, our item analysis shows that, even though the G8 score is a recommended and evaluated screening tool in oncologic patients, it has several relevant drawbacks in urologic oncology. First, the threshold for some items is very low. Second, the G8 score is mainly based on nutritional items, which are, according to our data, less relevant in urologic oncology. Third, several of the items are patient- and/or investigator-dependent because of their subjective nature. We therefore recommend that research should continue to improve the current screening tools in urologic oncology for geriatric frailty. One potential solution, which was proposed by Martinez et al., is a modified G8 score that is less focused on nutrition, reduces the threshold in polypharmacy, and includes additional aspects of daily living [10].

As the major limitation of our results and experiences, it must be noted that the results of the G8 screening scores were not systematically evaluated for each patient by a full geriatric assessment. However, we still consider our experience with the G8 score worth reporting, as it provides practical insight to the clinician on how the test is truly designed and how it functions in everyday practice. To our knowledge, this has never been done and published in urologic oncology before.

## Data Availability

The data that support the findings of this study are available from the corresponding author, [M.H.], upon reasonable request.
